# Blocked Filter of Anti-Embolic Device During Carotid Artery Stenting: A Rare Occurrence Posing Challenging Diagnostic Dilemma

**DOI:** 10.7759/cureus.19219

**Published:** 2021-11-02

**Authors:** Sarvesh C Mishra, Vivek Singh, Aviral Gupta, Srishti Sharma, Lavanya Tyagi

**Affiliations:** 1 Radio-Diagnosis, Sanjay Gandhi Post Graduate Institute of Medical Sciences, Lucknow, IND; 2 Pathology, Sanjay Gandhi Post Graduate Institute of Medical Sciences, Lucknow, IND; 3 Obstetrics and Gynecology, Javitri Hospital and Test Tube Baby Centre, Lucknow, IND

**Keywords:** anti-embolic device, case report, blocked filter device, dissection, carotid artery stenting

## Abstract

The use of anti-embolic devices (AED’s) is a common practice in carotid artery stenting (CAS). It prevents the passage of blood clots and thrombi generated during the procedure from embolizing into the intracranial circulation. Disadvantages include the passage of small particles and complications related to advancement, deployment, and recovery of the filters. The filter of the AED can get clogged due to the high load of the emboli generated during CAS causing a slowing of the intracranial blood flow which normalizes once the filter is removed.

Here, we are presenting a case of the filter of AED getting blocked due to entrapped thrombi or blood clots and mimicking dissection and, sharing our experiences associated with the event.

## Introduction

Atherosclerotic plaques causing narrowing in the ICA can rupture and migrate distally into the cerebral circulation leading to ischemic stroke in ~10% to 15% cases [1,2]. Both carotid artery stenting (CAS), as well as carotid endarterectomy (CEA), are viable treatment options in such cases and also in asymptomatic patients on medical therapy. Both these treatment options have their merits and demerits. CAS is quicker, less invasive than CEA, and does not require general anesthesia. Patients having medical conditions which pose a high risk for surgery are an indication for CAS [3]. Complications such as myocardial infarction, cranial nerve injury, cervical hematoma, wound infection, venous thromboembolism, perioperative hypertension, etc., are unlikely with CAS. However, periprocedural strokes (~4.1% against 2.3% in patients undergoing CEA) are more commonly seen in patients undergoing CAS [4]. This is attributed to the increased risk of distal cerebral thromboembolism associated with CAS. The embolic protection devices (EPDs) are used to prevent distal embolization into the cerebral circulation. However, they are not free of complications themselves. They are associated with difficulty in advancement or deployment across a stenotic segment and difficulty in retrieving the filter of the device. However, very rarely does the filter of the distal EPDs can get blocked by an embolus from plaque rupture during the CAS. This can give a very confusing imaging appearance on digital subtraction angiography (DSA) mimicking dissection similar to the one seen in our case. The filter of the AED can sometimes get clogged due to the high load of emboli leading to a slowing of the intracranial blood flow. We are sharing our experience from the case and also discussing the role of imaging modalities in plaque characterization prior to the procedure helping us in identifying vulnerable plaques. This eventually helps in better patient selection and counseling.

## Case presentation

**Figure 1:**
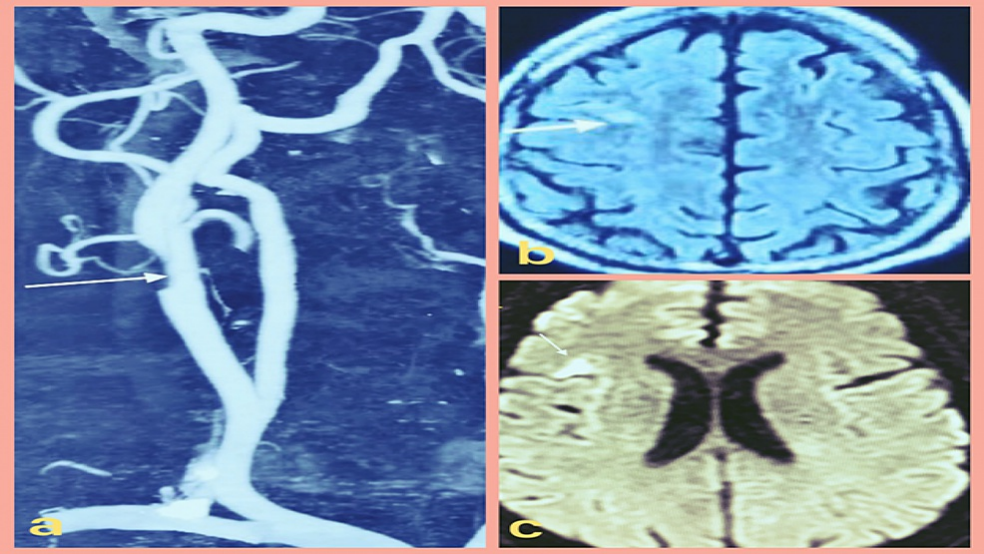
(a) CTA image showing a significant luminal narrowing in the right proximal ICA (denoted by white arrow). (b) Axial T2 fluid attenuation inversion recovery image showing a small acute infarct in the right frontal lobe (denoted by white arrow) appearing bright on (c) axial DWI image (denoted by white arrow). CTA: computed tomography angiography, ICA: internal carotid artery, DWI: diffusion-weighted imaging.

**Figure 2:**
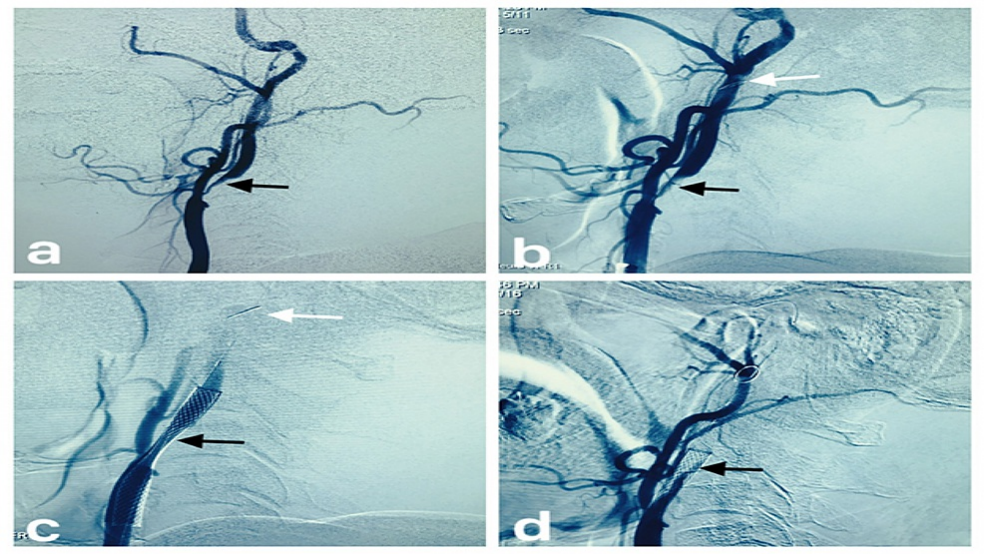
(a) The right CCA angiogram showing marked narrowing (denoted by black arrow) at the left proximal ICA. (b) The right CCA angiogram showing mild recanalization (denoted by a black arrow) of the narrowed segment after balloon angioplasty. The filter of the AED can be seen in the ICA distally (denoted by a white arrow). (c) The right CCA angiogram showing a stent with residual narrowing (denoted by a black arrow) of stenotic segment with AED filter seen distally (denoted by white arrow). (d) The right CCA angiogram post-intrastent ballooning showing trickle of contrast opacification across the posterior portion of the stent (denoted by a black arrow). CCA: common carotid artery, ICA: internal carotid artery, AED: anti-embolic device.

A 60-year-old hypertensive and the diabetic male patient presented in the neurology department with complaints of episodic loss of consciousness and fall. He also had a history of a few episodes of muscle weakness with associated slurring of speech in the past year which typically recovered within a few hours without residual neurological deficit. No history of associated visual disturbances or lateralization of weakness to one-half of the body. He had a history of embolic stroke in the right frontal lobe six months when he was admitted to some other hospital where computed tomography angiography (CTA) was done which revealed ~90% right proximal internal carotid artery (ICA) narrowing due to atheromatous plaque (Figure 1a). An echocardiogram was also done to rule out atrial fibrillation. Magnetic resonance imaging (MRI) head showed a small acute infarct in the right frontal lobe (Figure 1b-1c) and Carotid Doppler showed an echo lucent atheromatous plaque with significant luminal narrowing (~73%) and hemodynamic changes at the plaque site. The patient was referred to Neuroradiology Department where the patient and imaging were evaluated and he was posted for CAS after achieving reasonable glycemic control (Baseline HbA1c was 9.2 and baseline fasting blood glucose level was 236 mg/dl) using insulin. The patient was on long-term antiplatelet (tablet Ecosprin-AV 75 twice daily) and had a history of undergoing coronary artery bypass grafting 10 years back. Vascular access was gained via the right femoral artery and the patient was heparinized with 3000 internal units of intra-arterial heparin. ICA angiogram showed a short segment tight stenosis (Figure 2a). AED was advanced across the stenotic segment and deployed in the cervical ICA without any problem and angioplasty of the stenotic segment of proximal ICA was done using a 3 mm balloon. An angiogram was taken after the deployment which showed persistent stenosis (Figure 2b). A 7 mm × 40 mm caliber wall stent was deployed (Figure 2c). Intra-stent balloon dilatation was done 5 mm × 20 mm balloon. An angiogram was taken which showed no contrast flow into the right ICA beyond the stent with a narrow stream of contrast seen tracking along the posterior wall of the stent (Figure 2d).

The angiographic appearance was akin angiographic appearance seen in dissection. Due to this confusing angiogram, we deployed a longer 7 mm × 50 mm stent within the already placed stent. Repeat angiogram showed persistent angiogram findings.

We did aspirations repeatedly by advancing the guiding catheter close to AED but to no avail as abnormal angiographic appearance persisted. Angiograms to look for cross-flow showed poor cross flow via the anterior communicating artery and minimal flow via the posterior communicating artery.

**Figure 3:**
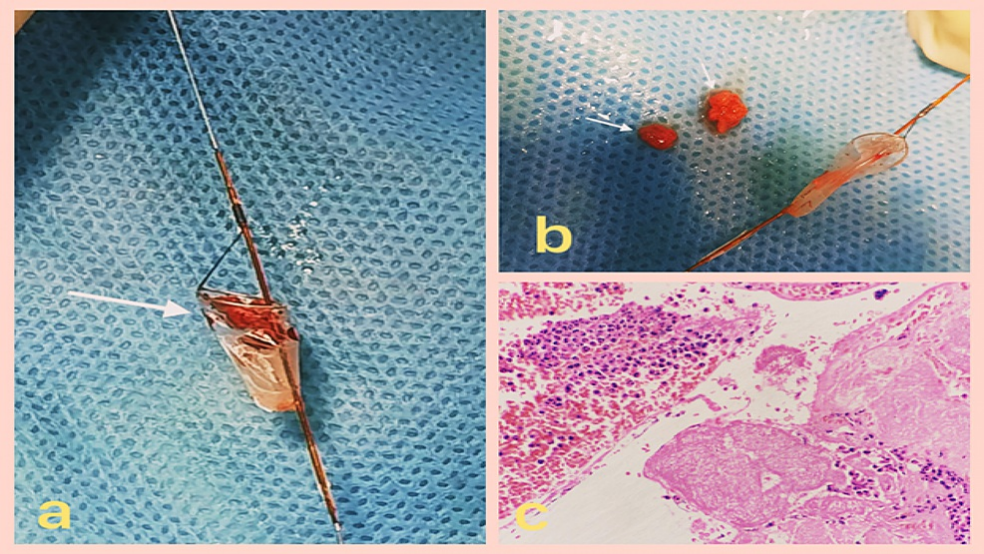
(a) Filter of the embolic device with trapped emboli; (b) emboli removed from the filter of the AED; (c) H&E, 400×, necrotic material along with hemorrhage and foci of neutrophilic infiltrate.

The patient started developing symptoms of cerebral ischemia evident as contralateral muscle weakness, decreased alertness, slurring of speech, and disturbed cognition. We decided to remove the filter as we suspected that we might be dealing with a case of thrombus entrapment within the filter. The AED was removed without any problem. The removed AED showed the blocked filter by an entrapped thrombus (Figure 3a). Angiogram showed patent stent and restoration of distal blood flow and the patient also improved symptomatically within a few minutes. The entrapped thrombus retrieved from the filter (Figure 3b) was sent for histopathological examination which showed necrotic material along with hemorrhage and foci of neutrophilic infiltrate (Figure 3c).

## Discussion

Intraprocedural cholesterol atheroembolization is one of the key elements responsible for poor outcomes after endovascular procedures [5]. This can be prevented by the use of anti-embolic devices (AED’s) also known as embolic protection devices or EPD’s in CAS. However, their use is not free of complications. We have come across complications of using filter distal AED’s like the passage of small particles and complications related to advancement, deployment, and recovery of the filters. However, a blocked filter of AED is a first for us. In our case, the AED effectively stopped the distal migration of the embolus. However, the imaging appearance due to a blocked filter was our first experience like this. Through this case, we want to create awareness about this dilemma which many might face if not aware of this imaging appearance.

Ultrasound, CT, and MRI can be used for identifying vulnerable plaques prior to CAS thus helping appropriate patient selection and counseling as these plaques are prone to thrombo-embolism during CAS.

The plaque morphology is an important factor in determining the risk of plaque rupture during CAS can be assessed prior to the procedure. Traditionally, the presence of echo lucent plaques on ultrasound has been shown to be associated with a higher incidence of ischemic complications following CAS [6]. Biasi et al. in their Imaging in Carotid Angioplasty and Risk of Stroke study (ICAROS) used a quantitative computer-assisted index of echogenicity grey-scale median (GSM) and concluded that both plaque echogenicity and degree of stenosis are independent factors determining the risk of plaque rupture and embolism [6]. A GSM value of >25 was associated with a low risk of an embolic event during CAS with low rates of stroke, death, and transient ischemic attack. They also suggested the use of a proximal embolic protection device in plaques with GSM <25 as the crossing of the stenosis itself has an inherent risk of embolization.

CTA can also be used in the evaluation of carotid artery plaque characteristics and risk stratification for peri-procedural embolization. Peripheral position of calcification in plaque is an independent risk factor for intra-operative and post-operative embolization [7]. Vascular wall calcification is associated with an increased risk of cardiovascular events and mortality three to four times [8]. Increased calcium load in a plaque poses difficulties in positioning the stent, dilatation of the stenotic segment, and required stent expansion increasing the risk of embolization [9]. Carotid plaque enhancement, ulceration of the plaque surface, and plaque ulceration are risk factors for embolization post-CAS [7].

MRI can also be used to assess plaque morphology and identify vulnerable plaques thus helping in risk stratification and allowing for better patient selection. Signal intensity on carotid artery MRI is used for evaluation in clinical practice [10,11] which is useful for identifying high-risk lesions [12,13]. This is made possible due to recent technical gains in the hardware of MRI. Intra-plaque hemorrhage which manifests as a T1 hyperintense signal is seen in vulnerable plaques and its presence is a high-risk factor in both symptomatic [14] and asymptomatic patients [15]. Plaque vulnerability also depends on its volume and morphology [16]. Expansive plaque remodeling is another factor associated with an increased risk of an ischemic event.

## Conclusions

Vulnerable plaques can be identified prior to the CAS using USG, CT, or MRI which helps in better patient selection and allows us to counsel the patient and the attendants in a better way. Vulnerable plaques can rupture during the CAS and can cause many complications due to thrombo-embolism. One such very rare complication is blocking the filter device of AED which can mimic dissection. This imaging appearance should be kept in mind in case there is no flow distal to the site of stenosis after the dilatation of the stenotic segment of the artery.
